# A novel approach to adenine-induced chronic kidney disease associated anemia in rodents

**DOI:** 10.1371/journal.pone.0192531

**Published:** 2018-02-07

**Authors:** Asadur Rahman, Daisuke Yamazaki, Abu Sufiun, Kento Kitada, Hirofumi Hitomi, Daisuke Nakano, Akira Nishiyama

**Affiliations:** Department of Pharmacology, Faculty of Medicine, Kagawa University, Kagawa, Japan; Tokushima University Graduate School, JAPAN

## Abstract

To date, good experimental animal models of renal anemia are not available. Therefore, the purpose of this study was to establish a novel approach to induce chronic kidney disease (CKD) with severe anemia by oral administration of adenine in rodents. Adenine was administered to 6-week-old male C57BL/6 mice (25 and 50 mg/kg body weight) by oral gavage daily for 28 days. Serum creatinine and BUN as well as hematocrit, hemoglobin (Hb) and plasma erythropoietin (EPO) levels were monitored to assess renal function and anemia, respectively. Adenine at 25 mg/kg for 28 days slightly increased plasma creatinine levels, but did not induce anemia. In contrast, 50 mg/kg of adenine daily for 28 days showed severe renal dysfunction (plasma creatinine 1.9 ± 0.10 mg/dL) and anemia (hematocrit 36.5 ± 1.0% and EPO 28 ± 2.4 pg/mL) as compared with vehicle-treated mice (0.4 ± 0.02 mg/dL, 49.6 ± 1.6% and 61 ± 4.0 pg/mL, respectively). At the end of experiment, level of Hb also significantly reduced in 50 mg/kg adenine administration group. Remarkable histological changes of kidney tissues characterized by interstitial fibrosis and cystic appearance in tubules were observed in 50 mg/kg of adenine treatment group. These results have demonstrated that oral dosing with adenine at 50 mg/kg for 28 days is suitable to induce a stable anemia associated with CKD in mice.

## Introduction

Anemia is a common hallmark of chronic kidney disease (CKD) which is associated with reduced quality of life and increased cardiovascular disease (CVD), hospitalizations, cognitive impairment and mortality [1]. It has also been shown that anemia is an independent predictor for ischemic cardiac events [2]. Therefore, anemia plays an important role in adding a further layer of complexity to the relationship between CKD and CVD [3]. Anemia management through recombinant human erythropoietin (rhEPO) or erythropoiesis stimulating agents (ESAs) greatly benefits patients with CKD by improving their debilitating symptoms, and relieving them from dependence on blood transfusions [4]. However, the current management and therapeutic approach for patients with renal anemia is controversial due to increased morbidity and mortality [5–7]. Therefore, there is a desperate need for studies to determine the precise mechanism of renal anemia in order to develop novel therapies or therapeutic strategies.

Animal models of CKD are important means of enabling translational research for investigating the pathophysiology of renal anemia and providing opportunities to assess the screening of potential novel therapies [8]. A number of animal models are available for induction of progressive renal failure including 5/6 nephrectomy (subtotal nephrectomy) and unilateral ureteral obstruction (UUO). However, these surgical induction of CKD in animals have numerous limitations such as a significant mortality rate and less anemia [9]. The adenine-induced CKD animal model has gained attention due to its relative ease of implementation without surgery and encouraging outcomes. Orally administered adenine metabolizes to 2,8-dihydroxyadenine, which forms crystal in the proximal tubular epithelia leading to inflammation and subsequent tubulointerstitial fibrosis [10, 11] as well as anemia [12]. In rodents, a number of studies have modified the original protocol developed by Yokozawa et al. [13] by changing the concentration of adenine in feed and/or the duration of administration to mimic renal anemia in humans [14–18]. However, administration of dietary adenine has a limitation: food intake is decreased significantly after mixing adenine with chow [19]. Therefore, food intake was not equal among the animals, and variability in the induction of CKD and/or anemia was obvious [18, 20].

The purpose of the present study is to establish a novel method of adenine administration in rodents to stably induce renal anemia. To avoid the variability of the effect of adenine, we measured the body weight of mice daily and administered the calculated amount of adenine accurately by oral gavage. There are few reports that have examined the effects of oral gavage of adenine in rats; however, no information is available regarding anemia [21]. Accordingly, we also followed a previous report [21] to induce CKD in rats and investigated the subsequent anemia.

## Materials and methods

### Animals

All experimental procedures in this study involving animals were carried out according to the ethical standards of Kagawa University, Japan and the principles of the Declaration of Helsinki (S1 File). The protocol of this study was reviewed and approved by the local institutional committee at Kagawa University, Japan. Five-week-old C57BL/6 mice and Wistar rats were purchased from Japan SLC Inc. (Shizuoka, Japan). Male rodents were preferred in this study because female rodents are resistant to kidney injury [22, 23] and less likely to develop adenine-induced CKD [24]. Animals were housed in specific-pathogen-free animal facilities under controlled temperature (24 ± 2°C) and humidity (55 ± 5%) conditions with a 12-hour light-dark cycle, and were given standard chow and had access to water *ad libitum*.

### Drugs

Adenine was purchased from Sigma-Aldrich, Inc. (Saint Louis, MO, USA). Carboxymethyl cellulose (CMC) was purchased from Wako Pure Chemical Industries Ltd. (Osaka, Japan).

### Experimental protocols

Following one week of acclimatization, 6-week-old C57BL/6 mice and Wistar rats were divided into three groups each (S1 Fig) based on the baseline plasma levels of creatinine and hematocrit data. In the control group, 0.5% CMC was administered by oral gavage to both mice (0.2 mL per 20 g body weight; n = 20) and rats (2 mL per 200 g body weight, n = 20). Adenine was administered to mice at 25 (n = 30) and 50 (n = 30) mg/kg body weight in 0.5% CMC by oral gavage (because mice were reluctant to consume adenine-containing chow) daily for 28 days. Furthermore, in rats, adenine at 200 (n = 30) and 600 (n = 30) mg/kg body weight in 0.5% CMC was administered by oral gavage daily for 10 days. During the observation period, at each time point 5–6 animals were euthanized with sevoflurane via precision vaporizer to measure renal function and anemia related parameters and the remaining animals were euthanized at the end of the observation period. In addition, to confirm this adenine-induced renal injury model is suitable to study renal anemia, rhEPO (5 IU) was injected subcutaneously every 2 days for 35 days in mice, after 28 days oral administration of 50 mg/kg adenine with an additional 7 days of recovery.

### Sample collection

Blood samples were collected from the tail vein to measure hematological parameters both in mice and rats. We collected blood samples after the end of adenine administration, as well as at various time points during the observation period (S1 Fig).

### Biochemical and hematological parameters

Plasma creatinine was measured by a commercially available kit (LabAssay Creatinine, Wako Pure Chemical Industries Ltd., Osaka, Japan). For hematocrit measurement, blood was collected in hematocrit tubes followed microhematocrit centrifugation. The length of the column of packed red cells was measured, divided by the length of the column of whole blood and multiplied by 100%. Levels of plasma BUN and hemoglobin (Hb), as well as total protein in urine were measured by an automated analyzer (7020-Automatic Analyzer; Hitachi High-Technologies, Tokyo, Japan). Plasma erythropoietin (EPO) level was measured in both mice and rats by ELISA (Epo Mouse ELISA Kit, Cat. No. KA1998, Abnova, Tapei City, Taiwan; Legend Max Rat Erythropoietin ELISA Kit, Cat. No. 442807, BioLegend, San Diego, CA, USA, respectively) in accordance with the manufacturers’ instruction. Levels of ferritin and gamma glutamyltransferase (γ-GT) were also measured in rat plasma by commercially available ELISA kits (Ferritin (Rat) ELISA kit, Cat. No. KA1949, Abnova; Rat γ-GT1 ELISA Kit, Cat. No. E-EL-RO404, Elabscience, Wuhan, China, respectively).

### Histological analysis

Following euthanasia of the mice and rats, kidneys were collected and perfused with 0.9% saline and then pieces of kidney were fixed in 10% neutral-buffered formalin, dehydrated in a concentration gradient of ethanol, cleared with xylene and embedded in paraffin. Tissue samples were cut into 5-μm-thick sections and stained with azan, as described previously [25].

### Gene expression in renal tissue

RNA was isolated from renal cortical tissues of rats by the phenol–chloroform extraction method and cDNA prepared as described previously [26]. The mRNA expression of α*-smooth muscle actin* (*Sma*) and *fibroblast growth factor (Fgf) 23* was analyzed by real-time PCR using an ABI Prism 7000 system with Power SYBR Green PCR Master Mix (Applied Biosystems, Foster City, CA, USA). The oligonucleotide primers for rats used were as follows (forward and reverse, respectively): 5′-ACGGCGGCTTCGTCTT CT-3′ and 5′-CCAGCTGACTCCATGCCAAT-3′ for *α-Sma* [27]; 5′-TTGGATCGTATC ACTTCAGC-3′ and 5′-TGCTTCGGTGACAGGTAG-3′ for *Fgf23* [28]; 5′-CCCTG GCTCCTAGCACCAT-3′, and 5′-CCTGCTTGCTGATCCACAT CT-3′ for *β-Actin* [27]. The abundance of target genes were normalized to *β-Actin* (as internal control) and analyzed by the 2^−ΔΔCt^ method as described previously [29].

### Statistical analysis

Data are the mean ± SEM. One-way analysis of variance followed by Dunnett’s multiple comparison test was used for all cross sectional one-factor data (plasma BUN, Hb, ferritin, γ-GT, urine protein, mRNA expression). Longitudinal (body weight, plasma creatinine, hematocrit and EPO) data were analyzed by two-way analysis of variance followed by the Bonferroni post-hoc test to determine differences between groups, except for mouse plasma creatinine and EPO data (one-way analysis of variance followed by Bonferroni post-hoc test). Data of the adenine administered rats and mice were compared with that of the respective vehicle-treated group. A P-value of P < 0.05 was considered statistically significant. Data were analyzed by GraphPad Prism 7 (GraphPad Software, Inc., CA, USA).

## Results

### High dose adenine treatment by oral gavage reduces body weight

**Fig 1:**
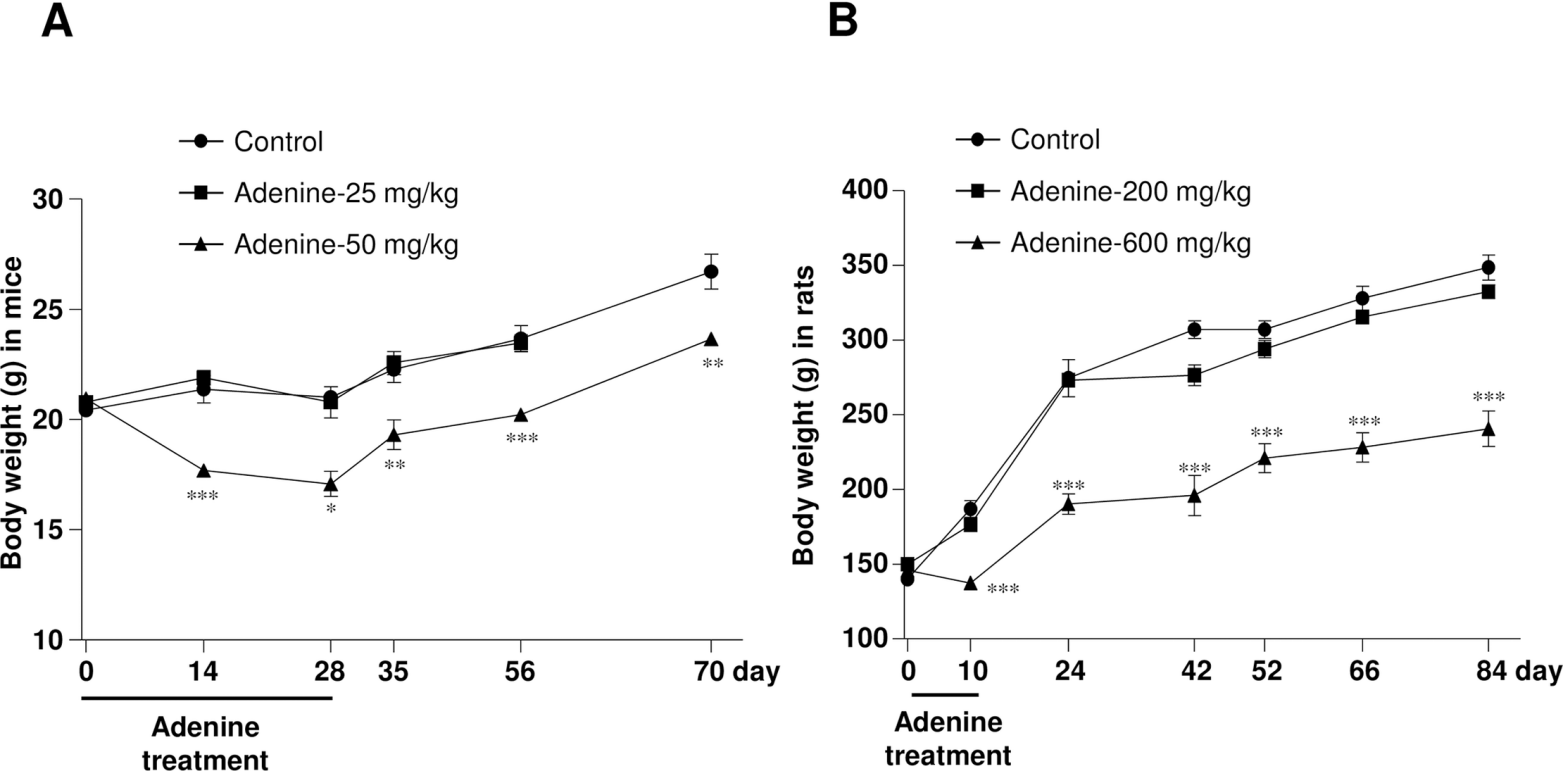
High dose of adenine by oral gavage reduces body weight in mice and rats. Changes of body weight during adenine administration and the observation period in (A) mice and (B) rats. * *P* < 0.05, ** *P* < 0.01, *** *P* < 0.001 vs. vehicle-treated mice or rats, respectively.

In C57BL/6 mice, the gradual increase in body weight in the vehicle-treated group signified a normal growth curve (Fig 1A). However, adenine at 25 mg/kg did not affect the body weight during or after administration as compared with vehicle-treated animals. In contrast, oral administration of adenine at 50 mg/kg reduced body weight significantly during and after 28 days of treatment.

In Wistar rats, adenine at 200 mg/kg did not affect body weight, while adenine at 600 mg/kg body weight for 10 days significantly decreased the body weight (Fig 1B). Food intake tended to increase at the end of experiments in both mice and rats (Table 1). Increase in body weight was accompanied by an increase in food intake during the observation period, up to 70 days in mice and 84 days in rats, respectively. However, body weight remained significantly lower in adenine-treated groups as compared with the respective vehicle-treated control animals.

**Table 1:** Plasma biochemical, hematological, metabolic and urinary parameters in rodents with adenine-induced renal injury.

Parameters	Mice				Rats			
	Day	Vehicle	25 mg/kg	50 mg/kg	Day	Vehicle	200 mg/kg	600 mg/kg
Plasma Creatinine (mg/dL)	28	0.3 ± 0.01	0.8 ± 0.1	1.8 ± 0.1***	10	0.5 ± 0.04	1.1 ± 0.1	3.02 ± 0.02***
	70	0.2 ± 0.03	-	1.2± 0.1**	84	0.7 ± 0.02	1.0 ± 0.06	2.3 ± 0.3***
Plasma BUN (mg/dL)	70	36.6 ± 1.1	-	73 ± 3.7***	66	24 ± 0.9	56 ± 2.2*	142 ± 1.9***
Hematocrit (%)	28	49.6 ± 1.6	48.6 ± 0.9	36.5 ± 1.0***	10	43.5 ± 2.2	41.7 ± 1.0	40.1 ± 1.2
	70	49.5 ± 0.7	-	33.9 ± 1.1***	84	43.4 ± 1.5	44.1 ± 1.7	30.1 ± 1.0***
Hemoglobin (g/dl)	70	16.8 ± 0.2	-	12.9 ± 0.2***	66	17.0 ± 0.1	14.2 ± 0.5	11.9 ± 0.5***
Total RBC (x10^4^/ μL)	-	-	-	-	763	763 ± 4.9	642 ± 20.3	-
Plasma EPO (pg/mL)	28	60.9 ± 4.0	44.8 ± 2.5	27.8 ± 2.3***	10	28.4 ± 3.7	7.5 ± 1.6***	2.0 ± 0.8***
	70	-	-	16.6 ± 4.7†††	84	28.6 ± 2.6	23.2 ± 3.4	13.1 ± 2.5**
Plasma ferritin (ng/mL)	-	-	-	-	66	238 ± 36	388± 29*	404 ± 42*
Plasma γ-GT (U/L)	-	-	-	-	66	3.4 ± 0.2	3.8 ± 0.1	4.3 ± 0.2
Food intake (mL)		4.8 ± 0.3	-	4.9 ± 0.2	66	13.0 ± 0.7	15.9 ± 0.6	14.3 ± 1.1
Water intake (mL)		5.9 ± 0.2	-	13 ± 0.4***	66	19.0 ± 1.1	38.8 ± 2.0	62.7 ± 4.1***
Urine volume (mL)	70	2.0 ± 0.2	-	7.9 ± 0.5***	66	11.7 ± 0.8	26 ± 2.1*	47 ± 3.5***
Urine protein (mg/day)	70	5.6 ± 0.5	-	9.7 ± 1.4*	66	5.7 ± 0.6	6.7 ± 1.0	24 ± 6.0***

Data are mean ± SE.

* *P* < 0.01

** *P* < 0.01

*** *P* < 0.001 vs. vehicle-treated mice or rats (corresponding time points) respectively

^†††^ *P* < 0.001 vs. vehicle-treated mice at 28 days.

### Adenine administration by oral gavage decreases renal function and manifests pathological features like CKD

**Fig 2:**
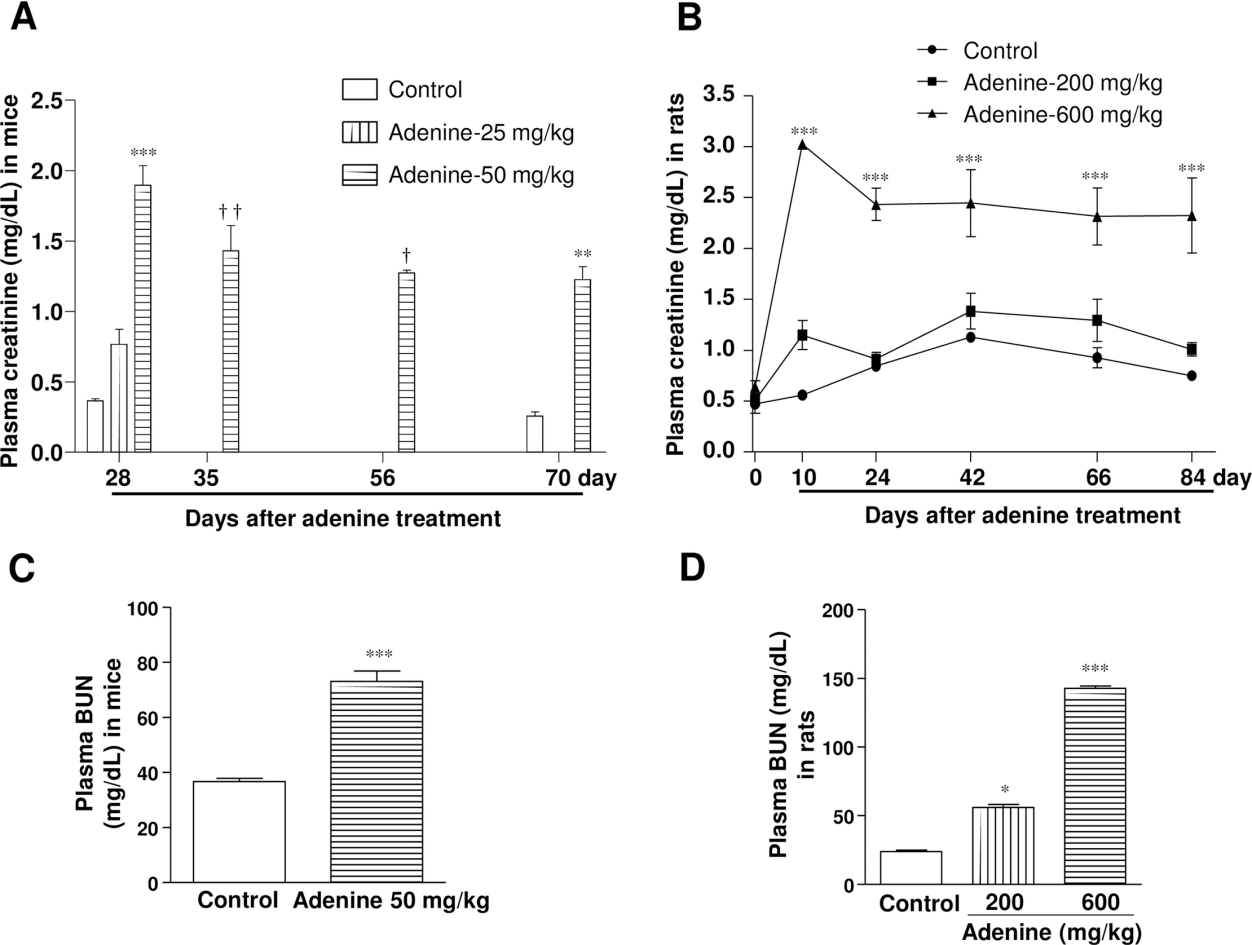
Administration of adenine by oral gavage decreases renal function. Changes in plasma creatinine levels after adenine administration and during the observation period in (A) mice and (B) rats. Levels of plasma BUN in (C) mice and (D) rats at day 70 and 66, respectively. * *P* < 0.05, ** *P* < 0.01, *** *P* < 0.001 vs. vehicle-treated mice or rats (corresponding time points) respectively; ^†^ *P* < 0.05, ^††^ *P* < 0.01 vs. vehicle-treated mice at 28 days.

In mice, oral administration of adenine at 25 mg/kg slightly, but significantly, increased plasma creatinine (0.8 ± 0.10 mg/dL) levels at day 28 (Fig 2A). In contrast, 50 mg/kg of adenine caused an approximately 5-fold increase in plasma creatinine levels (1.9 ± 0.10 mg/dL) as compared with the vehicle-treated mice (0.4 ± 0.02 mg/dL). During the observation period, levels of plasma creatinine tended to be decreased, but remained significantly higher in the 50 mg/kg adenine treatment group in comparison with the vehicle-treated mice at day 28. Consistently, BUN level in plasma also significantly higher in mice treated with adenine at 50 mg/kg at day 70 (Fig 2C).

In rats, adenine at 200 mg/kg caused a significant increase in plasma creatinine level (1.2 ± 0.14 mg/dL) compared with vehicle-treated rats (0.6 ± 0.05 mg/dL) (Fig 2B). However, the plasma creatinine level was elevated 5-fold (3.0 ± 0.02 mg/dL) in 600 mg/kg adenine-treated rats during the administration period. At day 24, plasma creatinine levels were approximately 4-fold higher and remained constant during the remainder of the observation period. Levels of plasma BUN levels in rats also significantly higher following administration of adenine at either 200 or 600 mg/kg at day 66 (Fig 2D).

Adenine administration at 50 mg/kg in mice and 600 mg/kg in rats caused a significant increase in urine protein excretion (Table 1), suggesting high doses of adenine induces proteinuria which is a pathological manifestation in patients with CKD. Moreover, plasma levels of γ-GT were significantly increased by adenine administration at 600 mg/kg in rats, indicating systemic toxicity and liver damage (Table 1).

### Adenine administration by oral gavage induces anemia

**Fig 3:**
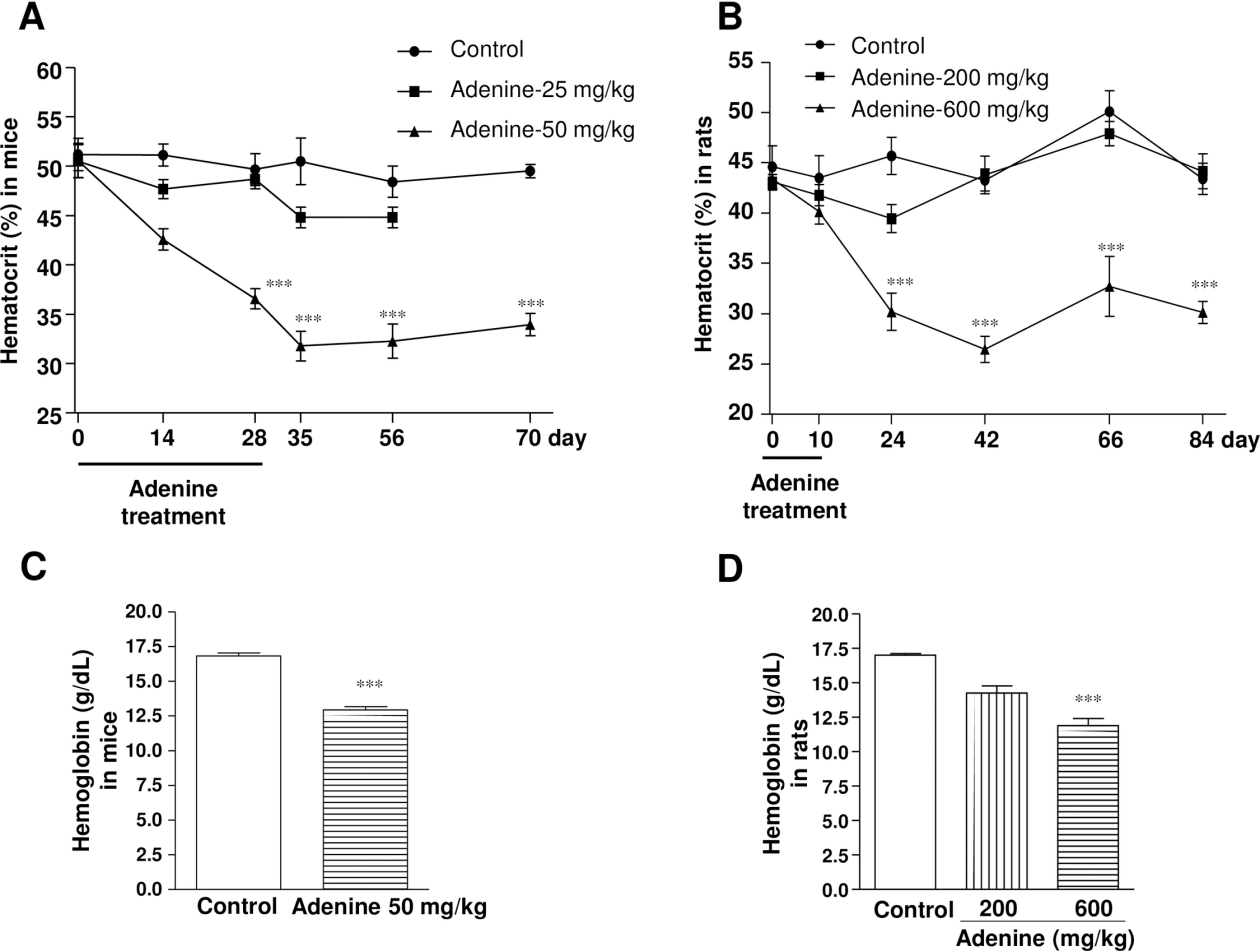
Administration of adenine by oral gavage reduces hematocrit and Hb levels. Changes in hematocrit levels after adenine administration and during the observation period in (A) mice and (B) rats. Levels of plasma Hb in (C) mice and (D) rats at day 70 and 66, respectively. ***P < 0.001 vs. vehicle-treated mice or rats (corresponding time points) respectively.

**Fig 4:**
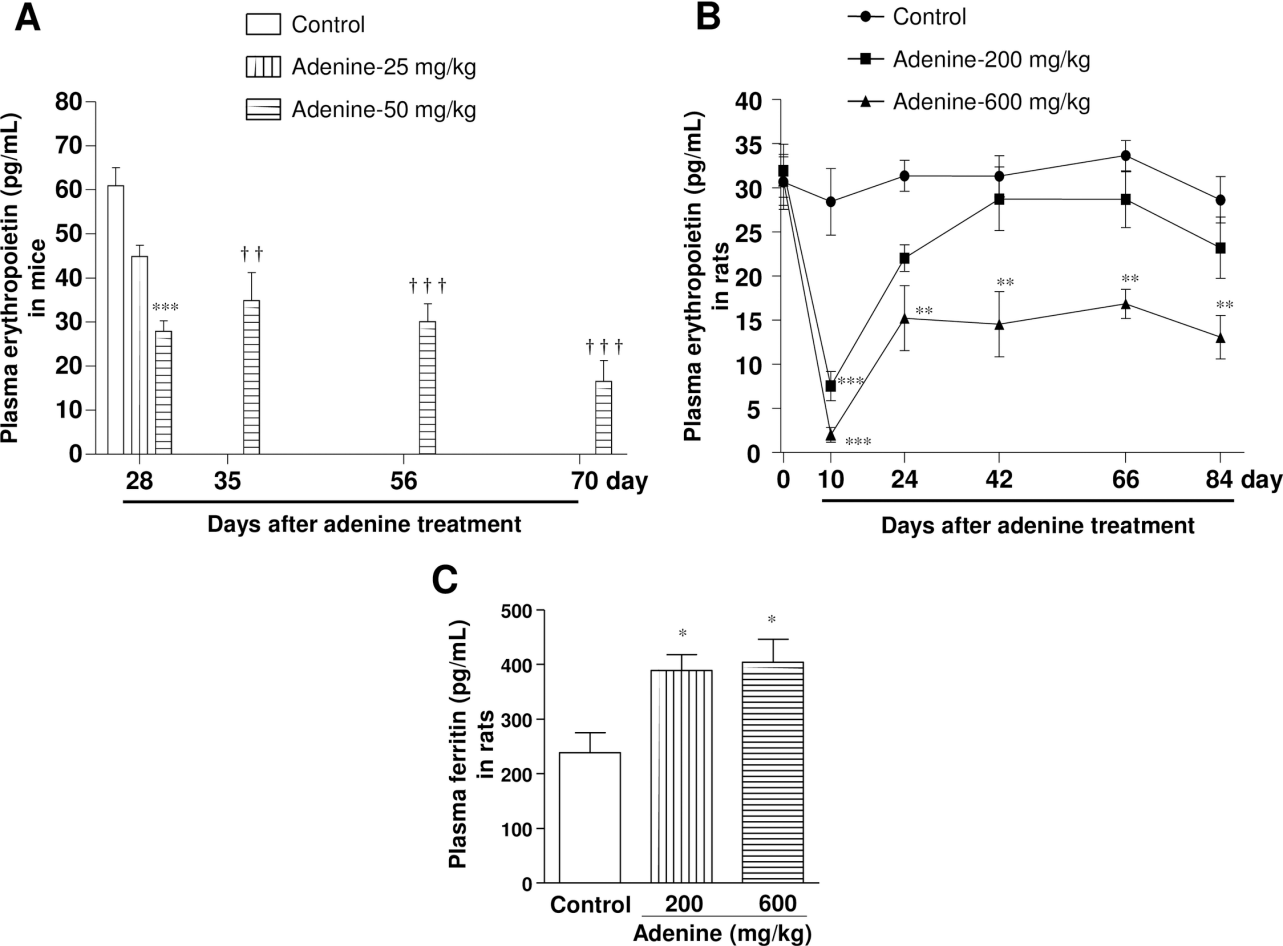
Administration of adenine by oral gavage impacts on plasma erythropoietin and ferritin levels. Changes in plasma erythropoietin (EPO) levels after adenine administration and during the observation period in (A) mice and (B) rats. Levels of plasma ferritin in (c) rats at day 66. * *P* < 0.05, ** *P* < 0.01, *** *P* < 0.001 vs. vehicle-treated mice or rats (corresponding time points) respectively; ^††^ *P* < 0.01, ^†††^ *P* < 0.001 vs. vehicle-treated mice at 28 days.

To observe anemia in CKD, hematocrit and plasma EPO levels were measured during and after the end of adenine administration in both C57BL/6 mice and Wistar rats (Figs 3 and 4). Adenine at 25 mg/kg in mice did not change the hematocrit (48.7 ± 0.9%) and EPO (45 ± 2.0 pg/mL) levels. However, adenine at 50 mg/kg administered for 28 days caused a significant reduction in hematocrit level (36.5 ± 1.0%) as compared with vehicle-treated mice (49.6 ± 1.6%), which remained significantly lower during the observation period (Fig 3A). Furthermore, plasma Hb levels were also significantly reduced in mice (16.8 ± 0.2 *vs*. 12.9 ± 0.2 g/dL) by oral administration of adenine at 50 mg/kg at day 70 (Fig 3C). Consistently, plasma EPO level was significantly reduced following adenine administration at 50 mg/kg for 28 days (28 ± 2 pg/mL vs. 61 ± 4 pg/mL), which also remained significantly lower upto the end of the experimental period (Fig 4A).

In rats, hematocrit level did not change during or after adenine administration at 200 mg/kg (Fig 3B). In contrast, adenine at 600 mg/kg caused a significant reduction in hematocrit level (30.2 ± 1.9%) at day 24 as compared with vehicle-treated rats (45.7 ± 1.7%), which remained significantly lower during the observation period. Consistently, plasma hemoglobin levels were significantly reduced (16.7 ± 0.1 vs.12.8 ± 0.6 g/dL) in rats following adenine administration at 600 mg/kg at day 66 (Fig 3D). Moreover, rat plasma EPO levels were significantly decreased by adenine administration at 200 or 600 mg/kg at day 10. However, in later period plasma EPO levels turned back to the normal range in 200 mg/kg adenine-treated rats, but remained significantly lower at 600 mg/kg adenine-treated group (Fig 4B). Plasma ferritin levels were significantly increased by oral administration of adenine at either 200 or 600 mg/kg in rats, which is a typical feature of CKD (Fig 4C).

### Adenine treatment changes the gross morphology of the kidney and induces damage and fibrosis to renal tissue

**Fig 5:**
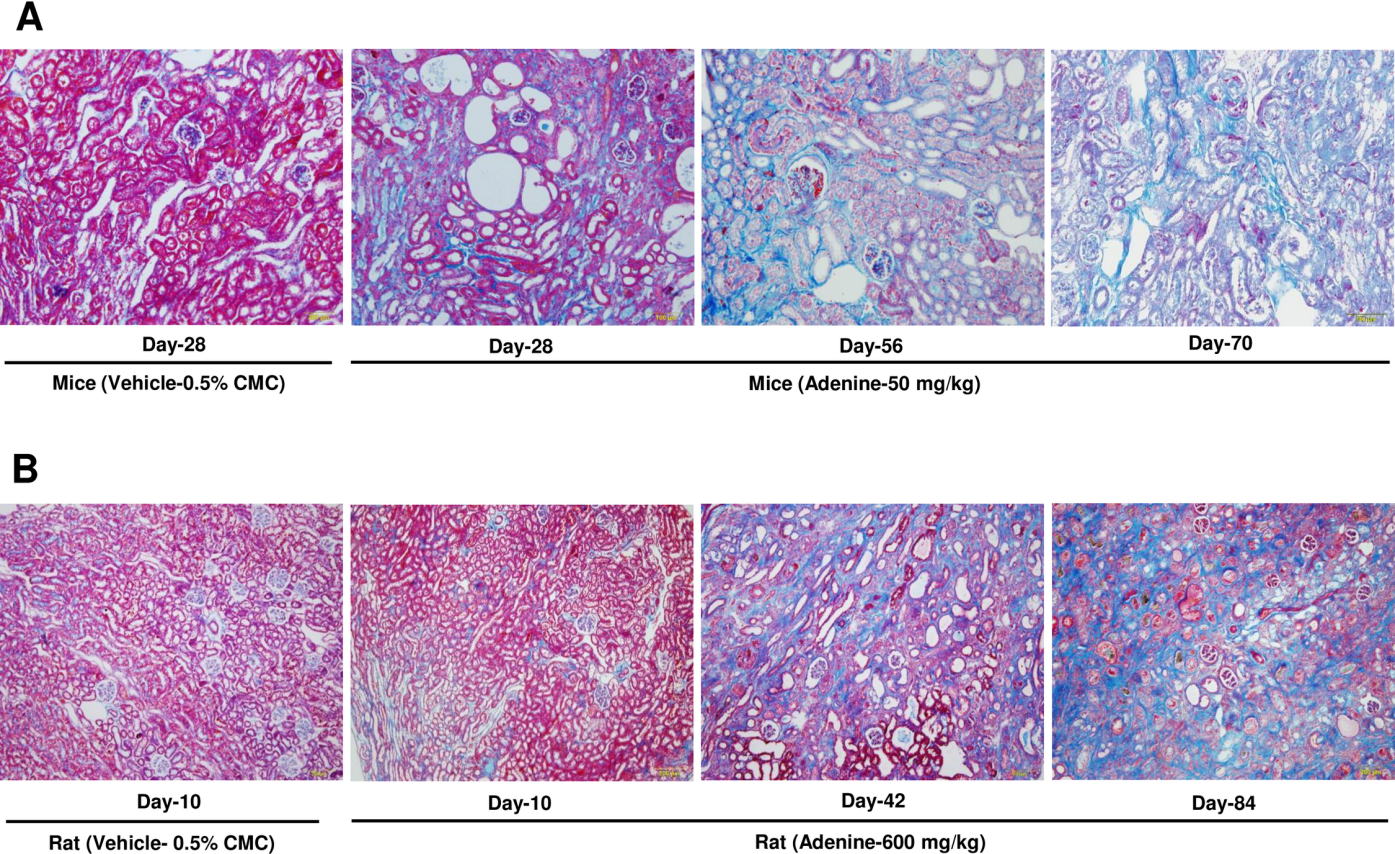
Adenine treatment via oral gavage induces tubulointerstitial fibrosis. Representative photomicrograph of azan staining of cortical sections of kidney tissues to demonstrate tubulointerstitial fibrosis in (A) mice and (B) rats after treatment with vehicle or adenine (50 mg/kg for mice and 600 mg/kg for rat) at different time points. Photomicrographs taken at 100× magnification.

In mice, swelling, discoloration and deformity of the kidney were more obvious after treatment with 50 mg/kg of adenine (S2A Fig). Azan staining of histological sections of mouse kidney tissue revealed severe tubulointerstitial fibrosis and cyst formation in kidneys after administration of adenine at 50 mg/kg during the observation period at days 56 and 70 (Fig 5A).

**Fig 6:**
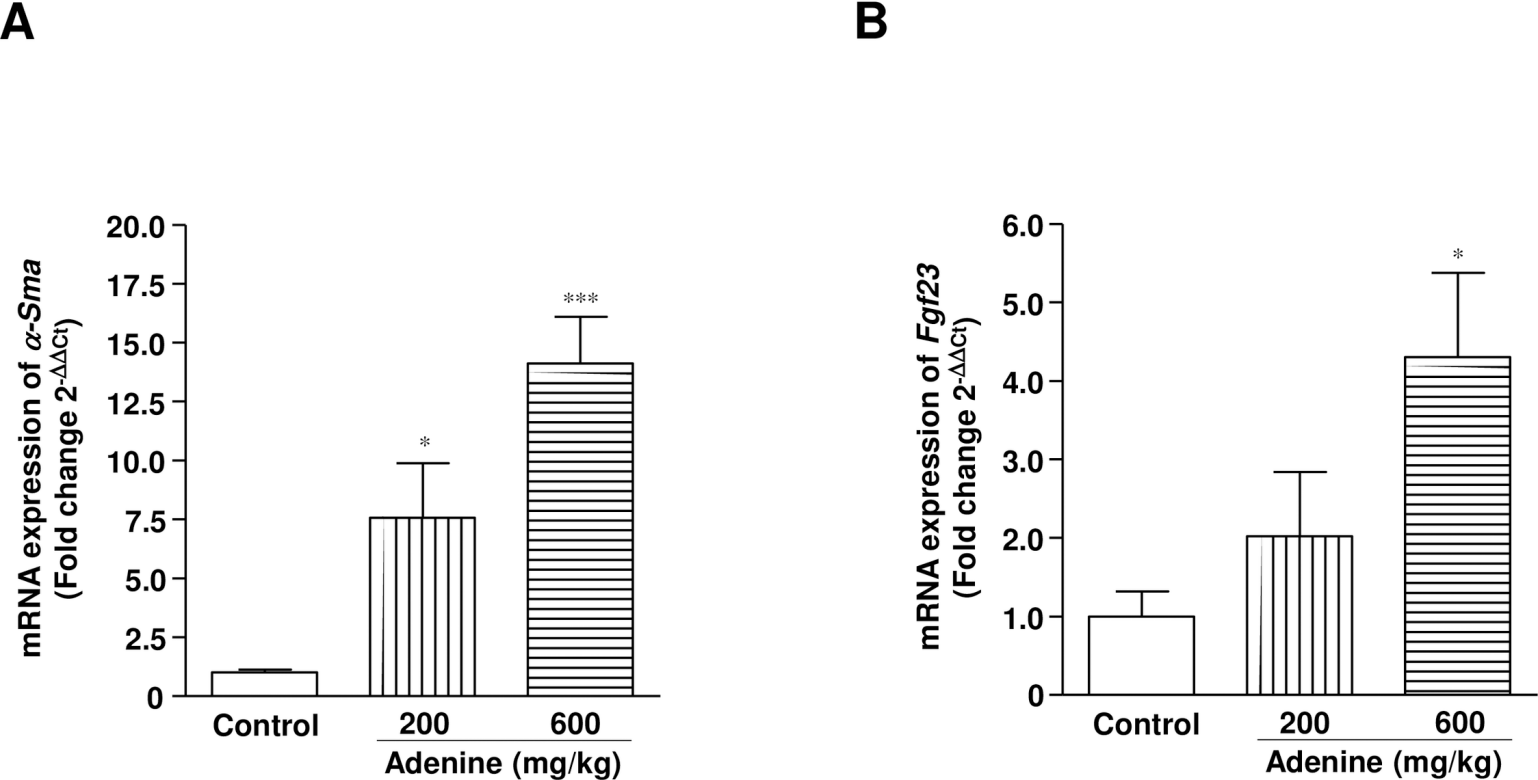
Oral administration of adenine increases the gene expression related to fibrosis and bone regeneration. Relative changes of mRNA expression of (A) *α-Sma* and (B) *Fgf23* in the renal cortical tissues of rats at day 84. * *P* < 0.05, *** *P* < 0.001 vs. vehicle-treated rats.

In rats, these gross morphological changes in the kidneys were also obvious in the 600 mg/kg adenine treatment group (S2B Fig). Severe tubulointerstitial fibrosis accompanied with cyst formation and tubular damage was obvious 42 and 84 days after oral administration of adenine at 600 mg/kg in rats (Fig 5B). Consistently, mRNA expression level of *α-Sma* (a critical mediator of fibrosis) was increased dramatically in cortical kidney tissues at day 84 (Fig 6A). Furthermore, *Fgf23* mRNA was expressed highly in the kidney tissues of rats treated with adenine at 600 mg/kg because it predicted CKD progression by interfering with bone remodeling (Fig 6B).

### Erythropoietin treatment improves adenine-induced CKD associated anemia in mice

**Fig 7:**
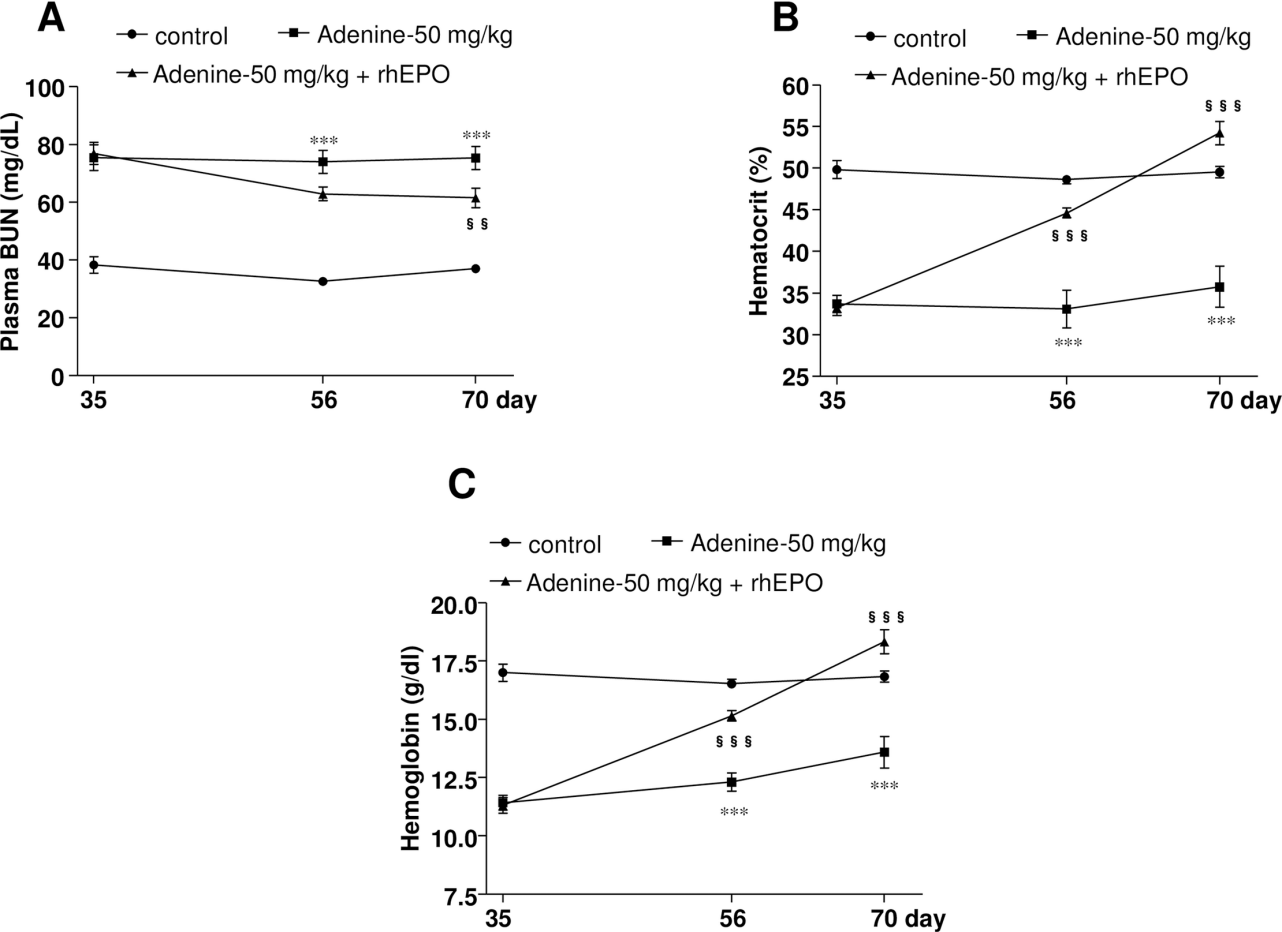
Erythropoietin treatment improves renal anemia in adenine-induced renal injury model. Plasma levels of (A) BUN, (B) hematocrit, and (C) Hb following subcutaneous injections of rhEPO (5IU) every 2 days interval for 5 weeks in mice with adenine-induced renal injury (50 mg/kg adenine for 4 weeks). *** *P* < 0.001 vs. vehicle-treated mice; ^§§^ *P* < 0.01, ^§§§^ *P* < 0.001 vs. adenine-treated (50 mg/kg) mice.

rhEPO treatment for 35 days caused a significant reduction in the plasma levels of BUN (Fig 7A) in mice. In contrast, levels of hematocrit (Fig 7B) and Hb (Fig 7C) gradually increased just after starting rhEPO treatment and significantly increased within 21 days of treatment and remained significantly higher at the end of the experiment. These data suggest that the adenine-induced renal injury model of rodents is a convenient tool to study anemia associated with CKD.

## Discussion and conclusions

To date, good experimental mouse models of renal anemia are not available. Here, we have established stable renal anemia in mice. We showed that adenine at 50 mg/kg administered through oral gavage for 28 days is enough to induce CKD with severe anemia in mice. Our data also demonstrate that biological variation among the animals in the adenine treated groups is less in comparison with the previously reported models of dietary administration of adenine [10, 20, 30].

In the initial report of adenine-induced CKD, adenine was given to rats mixed with feed at a concentration of 0.75% (w/w) for 4 weeks [11]. Many studies have followed the same protocol [23, 24] or modified the concentration of adenine in feed, e.g. 0.25% [14] and 0.5% [14, 16]. Duration of adenine administration has also been changed to obtain varying degrees of CKD with targeted pathophysiological conditions [30]. Dietary adenine administration at the concentration of 0.15–0.3% for 6–8 weeks has been reported to induce severe renal injury in C57BL/6 mice [17, 18, 31]. However, there is no available information regarding the anemia status in mice following adenine administration. We showed that adenine at 25 mg/kg did not change the hematocrit or plasma EPO levels after 28 days of oral administration, while these levels tended to decrease during the observation period. In contrast, 50 mg/kg oral administration of adenine for 28 days caused a significant reduction in hematocrit and plasma EPO levels as compared with the vehicle-treated mice, which remained significantly lower during the observation period of up to 70 days. In agreement with this, Hb levels also significantly reduced in mice at day 70. We also found that adenine at 100 mg/kg body weight was lethal for mice (data not shown). Recent studies have demonstrated that EPO treatment significantly reduced the BUN levels in renal ischemic reperfusion in rats [32] and 5/6 nephrectomy in mice [32, 33]. In the present study, we observed that rhEPO (5 IU) administration significantly decreased the plasma level of BUN, and increased the hematocrit and hemoglobin levels in adenine (50 mg/kg)-induced anemic mice, suggesting an improvement of renal function and anemia in these animals. Thus, our data revealed that adenine at 50 mg/kg induces severe anemia in mice, which mimics clinical renal anemia in humans, and this model is also suitable to study the pathophysiology and treatment of renal anemia.

Studies have shown that in Wistar rats, 0.75% (w/w) adenine diet for 4 weeks and an additional 4 weeks observation caused a severe anemia with hemoglobin levels of 7.6 ± 1.7 g/dL [30], and a hematocrit of 32.6±1.2% [34] (13.8±1.2 g/dL and 51.0±0.6% in control rats, respectively). However, Terai et al. [21] demonstrated the induction of renal disease by oral dosing of rats with 600 mg/kg adenine, although there was no reported information regarding anemia. In the present study, we showed that oral administration of adenine at 600 mg/kg for 10 days with an additional 14 days observation caused a significant reduction in hematocrit as well as plasma EPO levels, which remained at significantly lower levels than the controls for the 84 day observation period. A significantly lower level of Hb also confirmed the anemia in rats after administration of 600 mg/kg adenine. We also found that 200 mg/kg of adenine tended to decrease hematocrit and plasma EPO levels at day 24, but they soon returned to the normal level. In the present study, at day 10, both 200 and 600 mg/kg adenine caused a significant reduction in plasma levels of EPO in rats. Clinical studies have shown that acute renal injury stimulates EPO production at the beginning of the disease with a strong tendency to decrease just after the course of injury [35]. Similarly, renal HIF-1 was upregulated within 2 hours following acute renal injury and totally diminished within 24 hours [36]. This study also suggests that HIF induction is maximal with moderate hypoxia and declines with the increasing severity of hypoxia. In contrast, a recent study showed that 100 mg/kg adenine for 7 days caused severe renal cellular hypoxia in rats [37]. Collectively, a dramatic fall of EPO concentration at day 10 might be due to the reduction of expression of HIF-1α in adenine-induced severe cellular hypoxic kidneys in rats. Moreover, a recent clinical study has shown that renal anemia is caused by relative EPO deficiency [38], which is a consequence of injury and fibrosis as well as accumulation of myofibroblasts in the kidney [12]. In the present study, adenine administration in mice (50 mg/kg) and rats (600 mg/kg) induced severe tubulointerstitial fibrosis in the kidneys that was evident from the histomorphological changes by azan staining as well as increased mRNA level of α-*Sma* expression in cortical tissues. Consistently, Zhang et al. have demonstrated that adenine (200 mg/kg) given for 3 weeks in rats upregulated the expression of pro-fibrotic proteins such as *α-Sma*, collagen I, fibronectin, plasminogen activator inhibitor-1, and transforming growth factor-β1 significantly [39]. Expression of fibrotic proteins was not evaluated in the present study, but a similar level of kidney injury was observed in rats after adenine administration at 600 mg/kg for 10 days. Recently, it has demonstrated that the bone-derived hormone, FGF23 regulates systemic phosphate homeostasis, vitamin D metabolism and α-Klotho expression through a novel bone-kidney axis [40]. However, FGF23 levels increase gradually along with kidney injury, leading to reductions in 1,25(OH)2D levels and secondary hyperparathyroidism in individuals with CKD [41]. Interestingly, our results revealed that adenine administration at 600 mg/kg, but not 200 mg/kg caused significantly high expression of *Fgf23* mRNA that mimics the clinical condition of CKD. Therefore, all of these data have documented that 10 days of treatment with adenine at 200 mg/kg does not induce severe renal injury and anemia, while at 600 mg/kg caused a severe state of anemia in rats.

A number of studies have already shown changes in renal functional parameters in adenine-treated mice and rats [11, 15, 17, 18, 30, 42]. In mice, plasma creatinine was elevated by 1.5-fold in comparison with vehicle-treated mice following 0.2% dietary administration of adenine for 4 weeks [17]. However, our data revealed that plasma creatinine levels were increased by 5-fold following 50 mg/kg oral administration of adenine for 28 days in mice, which remained significantly higher during the observation period of up to 70 days. Consistent with previous data shown by Terai *et al*. [21], we also documented that 10 days of oral administration of adenine at 600 mg/kg body weight causes a significant increase in plasma creatinine levels in rats, which remained elevated during the observation period. Previous studies showed that 4 weeks adenine (0.75% w/w) administration through feed resulted in a significant increase in plasma creatinine [30] and decrease in creatinine clearance [15] in rats. Moreover, in the present study, plasma levels of BUN and protein excretion in urine were significantly increased in rats treated with oral administration of adenine at 600 mg/kg, suggesting an impairment of renal function. Furthermore, the mortality rate was 20% in the rats treated with 600 mg/kg adenine at the end of the experiment and the pathological manifestations were severe, which mimics the clinical features of CKD. We also showed that 200 mg/kg of adenine caused a transient increase in plasma creatinine during oral administration of adenine. However, these levels were not significantly different in comparison to the vehicle-treated rats after day 24. Importantly, increases in plasma creatinine levels in adenine-treated mice and rats were associated with renal tissue injury.

Previous studies have shown that dietary adenine administration (for 8 weeks in mice [43]; and for 3 weeks in rats [44]) induces renal injury, which is associated with a reduction in reticulocyte count, suggesting a hyporegenerative anemia. In the present study, the novel approach of adenine administration by oral gavage shortens the duration of renal injury induction (to 4 weeks in mice and 10 days in rats). However, the extent of renal injury and anemia are similar among both current and previous studies. These data suggest that adenine administration through oral gavage and diet induces renal anemia which is associated with hyporegenerative anemia. Water balance data indicated that water retention was significantly increased by adenine administration either at 600 mg/kg in rats or 50 mg/kg in mice, compared with the respective vehicle-treated animals [data not shown], suggesting that adenine-induced renal injury caused impairment in the excretion of water through urine. This water retention is also evident from hematocrit data, which is a characteristic feature of CKD [45]. We also observed that adenine at 600 mg/kg significantly increased plasma γ-GT in rats, consistent with a previous report of dietary adenine administration [15]. In contrast, 200 mg/kg adenine induced a significant reduction in the total RBC count without changing plasma γ-GT levels [Table 1]. Taken together, these data suggest that adenine-induced anemia is not predominantly induced by its toxic effect on erythropoiesis. Eryptosis, a dramatic loss of erythrocytes due to death of suicidal erythrocytes, is one of the important underlying mechanism of anemia in CKD as well as in end stage renal disease (ESRD) [46]. In clinical studies, it has been demonstrated that anemia is aggravated in CKD by eryptosis [47, 48] which is triggered by increased cytosolic Ca^2+^ concentration, oxidative stress and ceramide. Therefore, the severe anemia induced by adenine in rodents might also be associated with eryptosis.

Thus the present study has demonstrated that oral administration of adenine at 50 mg/kg for 28 days in mice or at 600 mg/kg for 10 days in rats induces a significant anemia, which is accompanied by renal tissue injury and dysfunction. In conclusion, we have established a novel approach to renal anemic models in both mice and rats. These animal models would be important and useful tools for translational research on renal anemia in humans.

## Supporting information

S1 Fig Experimental scheme. Oral administration of adenine at (A) 25 or 50 mg/kg body weight for 28 days in mice or (B) 200 or 600 mg/kg body weight for 10 days in rats. In mice the observation period was up to 70 days while in rats it was up to 84 days. Arrows (**↑**) indicate the blood collection time points.
(TIF)

S2 Fig Gross changes in kidney morphology after adenine treatment. Changes in size and color of kidney during the observation period in (A) mice and (B) rats after treatment with vehicle (0.5% CMC) or adenine (50 mg/kg for mice and 600 mg/kg for rat) at different time points.
(TIF)

S1 File The ARRIVE guidelines checklist. (DOCX)
