# Supplementary figures and images for: A novel approach to adenine-induced chronic kidney disease associated anemia in rodents
Source: PLoS One. 2018 Feb 7;13(2):e0192531. doi: 10.1371/journal.pone.0192531 (PMC5802942; doi:10.1371/journal.pone.0192531)

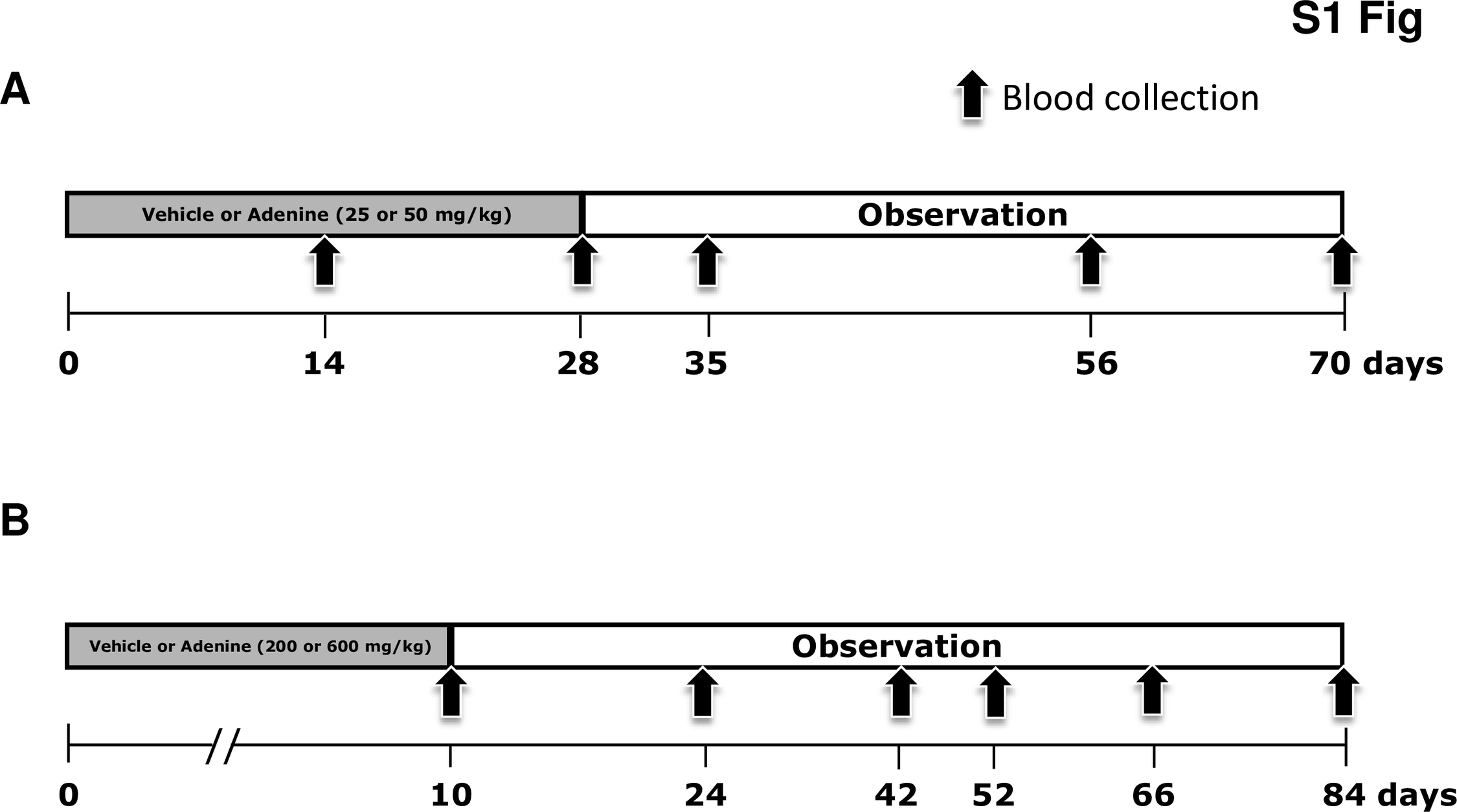

Supplement: S1 Fig — Oral administration of adenine at (A) 25 or 50 mg/kg body weight for 28 days in mice or (B) 200 or 600 mg/kg body weight for 10 days in rats. In mice the observation period was up to 70 days while in rats it was up to 84 days. Arrows (↑) indicate the blood collection time points. (TIF) [file pone.0192531.s001.tif]

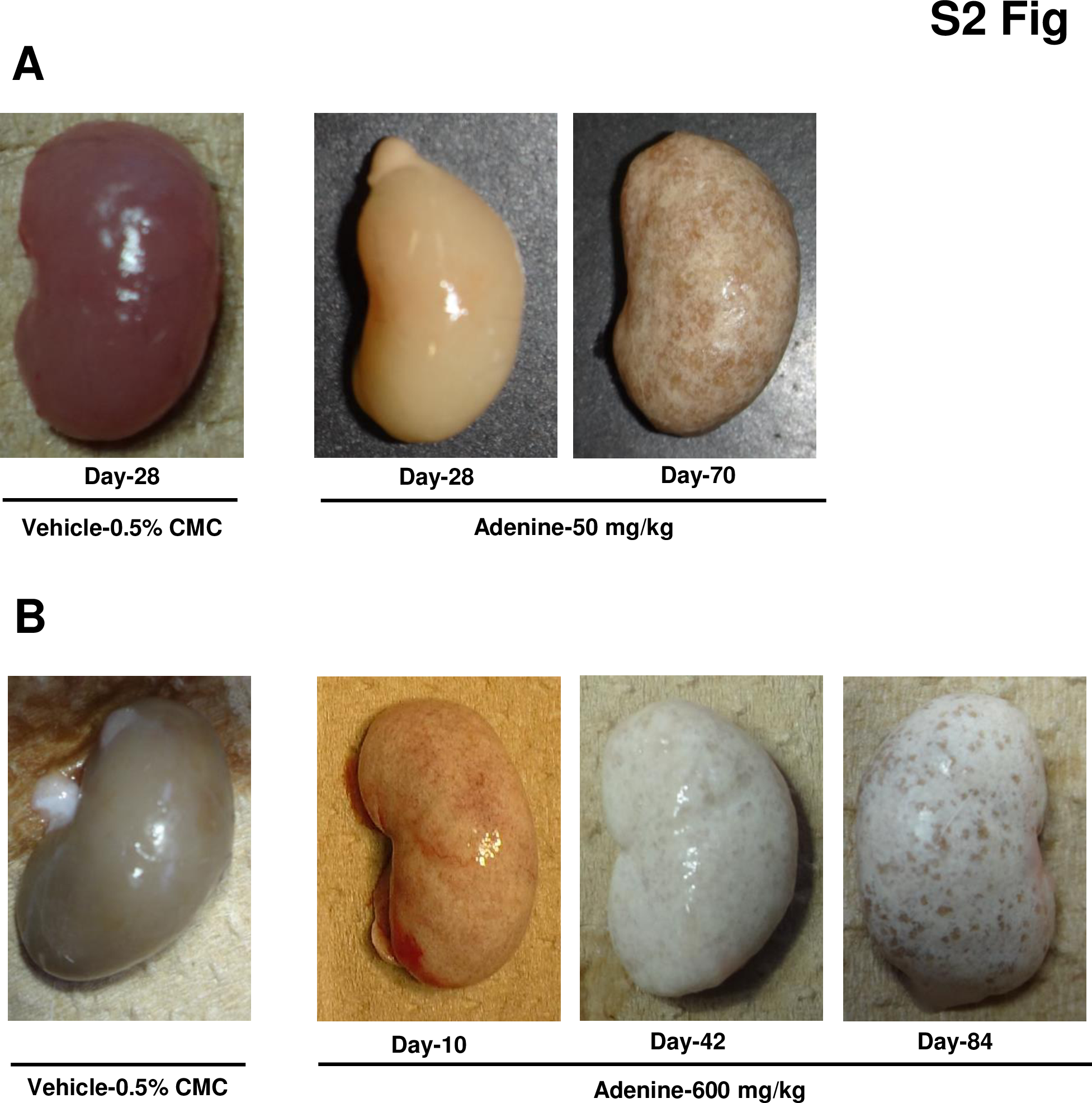

Supplement: S2 Fig — Changes in size and color of kidney during the observation period in (A) mice and (B) rats after treatment with vehicle (0.5% CMC) or adenine (50 mg/kg for mice and 600 mg/kg for rat) at different time points. (TIF) [file pone.0192531.s002.tif]
