# Supplementary material for: A novel approach to adenine-induced chronic kidney disease associated anemia in rodents
Source: PLoS One. 2018 Feb 7;13(2):e0192531. doi: 10.1371/journal.pone.0192531 (PMC5802942; doi:10.1371/journal.pone.0192531)

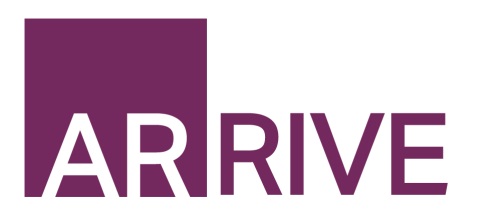


The ARRIVE Guidelines Checklist

Animal Research: Reporting In Vivo Experiments

Carol Kilkenny^1^, William J Browne^2^, Innes C Cuthill^3^, Michael Emerson^4^ and Douglas G Altman^5^

*^1^The National Centre for the Replacement, Refinement and Reduction of Animals in Research, London, UK, ^2^School of Veterinary Science, University of Bristol, Bristol, UK, ^3^School of Biological Sciences, University of Bristol, Bristol, UK, ^4^National Heart and Lung Institute, Imperial College London, UK, ^5^Centre for Statistics in Medicine, University of Oxford, Oxford, UK.*

|  | | ITEM | RECOMMENDATION | Section/ Paragraph |
| --- | --- | --- | --- | --- |
|  | \| Title \| 1 \| Adenine-induced chronic kidney diseases associated anemia in rodents. \| \| --- \| --- \| --- \| |  |  |  |
|  | \| Abstract \| 2 \| To date, good experimental animal models of renal anemia are not available. Therefore, the purpose of this study was to establish a novel approach to induce chronic kidney disease (CKD) with severe anemia by oral administration of adenine in rodents. Adenine was administered to 6-week-old male C57BL/6 mice (25 and 50 mg/kg body weight) by oral gavage daily for 28 days. Serum creatinine and BUN as well as hematocrit, hemoglobin (Hb) and plasma erythropoietin (EPO) levels were monitored to assess renal function and anemia, respectively. Adenine at 25 mg/kg for 28 days slightly increased plasma creatinine levels, but did not induce anemia. In contrast, 50 mg/kg of adenine daily for 28 days showed severe renal dysfunction (plasma creatinine 1.9 ± 0.10 mg/dL) and anemia (hematocrit 36.5 ± 1.0% and EPO 28 ± 2.4 pg/mL) as compared with vehicle-treated mice (0.4 ± 0.02 mg/dL, 49.6 ± 1.6% and 61 ± 4.0 pg/mL, respectively). At the end of experiment, level of Hb also significantly reduced in 50 mg/kg adenine administration group. A remarkable histological changes of kidney tissues characterized by interstitial fibrosis and cystic appearance in tubules were observed in 50 mg/kg of adenine treatment group. These results have demonstrated that oral dosing with adenine at 50 mg/kg for 28 days is suitable to induce a stable anemia associated with CKD in mice. \| \| --- \| --- \| --- \| |  |  |  |
|  | INTRODUCTION |  |  |  |
|  | \| Background \| 3 \| Animal models of CKD are important means of enabling translational research for investigating the pathophysiology of renal anemia and providing opportunities to assess the screening of potential novel therapies. Although a number studies demonstrated the adenine administration through feed to induce renal injury in rodents, however, dietary adenine administration has some limitation, specially the reduction of food intake after mixing of adenine with chow. Therefore, in the present study we approached the adenine administration through oral gavage to induce a stable renal injury and subsequent renal anemia in rodents. \| \| --- \| --- \| --- \| |  |  |  |
|  | \| Objectives \| 4 \| To establish a novel method of adenine administration in rodents to stably induce renal anemia. \| \| --- \| --- \| --- \| |  |  |  |
|  | METHODS |  |  |  |
|  | \| Ethical statement \| 5 \| All procedures were performed in this studies involving animals were in accordance with the ethical standards of Kagawa University, Japan and the principles of the Declaration of Helsinki. The protocol of this study was reviewed and approved by the local institutional committee at Kagawa University, Japan. \| \| --- \| --- \| --- \| |  |  |  |
|  | \| Study design \| 6 \| 1. Grouping For mice study, 1. Vehicle, 2. Adenine 25 mg/kg, 3. Adenine 50 mg/kg body weight. For rat study, 1. Vehicle, 2. Adenine 200 mg/kg, 3. Adenine 600 mg/kg body weight. 2. Grouping was done based on baseline parameters (plasma levels of creatinine and haematocrit data) 3. The experimental unit was the group of animals. \| \| --- \| --- \| --- \| |  |  |  |

The ARRIVE guidelines. Originally published in *PLoS Biology*, June 2010^1^

| \| Experimental procedure \| 7 \| 1. For mice study, in control 0.5% Carboxymethyl cellulose (CMC, 0.2 mL for 20 gm body weight) as vehicle and adenine at 25 and 50 mg/kg body weight with 0.5% CMC (0.2 mL for 20 gm body weigh); and for rat study, in control 0.5% CMC (2 mL for 20 gm body weight) and adenine 200 and 600 mg/kg body weight with 0.5% CMC (2 mL for 20 gm body weight) were administered through oral gavage. 2. Gavage was done every morning around 10 AM. 3. All experiments were performed in the animal laboratory. 4. Sevoflurane was used for euthanasia during sacrifice. \| \| --- \| --- \| --- \| |  | |  |
| --- | --- | --- | --- | --- | --- | --- |
| \| Experimental animals \| 8 \| 1. Five-week-old male C57BL/6 mice (around 20 gm) were used for mice study while five-week-old male Wistar rats (around 150 gm) for rat study 2. Animals were purchased from Japan SLC Inc. (Shizuoka, Japan). \| \| --- \| --- \| --- \| |  | |  |
| \| Housing and husbandry \| 9 \| 1. Animals were housed in specific-pathogen-free animal facilities 2. Under controlled temperature (24 ± 2°C) and humidity (55 ± 5%) conditions with a 12-hour light-dark cycle, 3. Given standard chow and had access to water ad libitum. \| \| --- \| --- \| --- \| |  | | |
| \| Sample size \| 10 \| 1. Total 80 mice and 80 rats were used. Twenty animals were used for vehicle groups and 30 for each adenine treatment group both in mice and rat studies. \| \| --- \| --- \| --- \| |  | | |
| \| Allocating animals to experimental groups \| 11 \| 1. Animals were allocated to experimental groups based on the baseline plasma levels of creatinine and haematocrit data. 2. First vehicle and then adenine were administered. \| \| --- \| --- \| --- \| |  | | |
| \| Experimental outcomes \| 12 \| In mice studies, only in 50 mg/kg adenine group 3 animals died during the adenine administration period, however, no animals died during the observation period. In contrast, 2 animals died during the observation period in 600 mg/kg adenine administration group in rat experiments. \| \| --- \| --- \| --- \| |  | | |
| \| Statistical methods \| 13 \| One-way analysis of variance followed by Dunnett’s multiple comparison test was used for all cross sectional one-factor data (plasma BUN, Hb, ferritin, γ-GT, urine protein). Longitudinal (body weight, plasma creatinine, hematocrit and EPO) data were analyzed by two-way analysis of variance followed by the Bonferroni post-hoc test to determine differences between groups, except for mouse plasma creatinine and EPO data (one-way analysis of variance followed by Bonferroni post-hoc test). \| \| --- \| --- \| --- \| |  | | |
| RESULTS |  | | |
| \| Baseline data \| 14 \| 1. In mice studies, 50 mg/kg adenine and in rat studies, 600 mg/kg adenine administration caused reduction of body weight during adenine administration, however it recovered during the observation period. \| \| --- \| --- \| --- \| |  | | |
| \| Numbers analysed \| 15 \| 1. During observation period in each time point we sacrificed 5-6 animals from each group for data collection and remaining animals were sacrificed during the end of experiments. So, for each time point we have analysed the data for 5/6 animals for each group. 2. Only the data for dead animals were excluded from each time point. \| \| --- \| --- \| --- \| |  | | |
| \| Outcomes and estimation \| 16 \| We used standard error for analysis. \| \| --- \| --- \| --- \| |  | | |
| \| Adverse events \| 17 \| a. In mice study, only for adenine at 50 mg/kg body weight, some animals showed dramatic reduction of body weight. However, this problem was recovered very soon. \| \| --- \| --- \| --- \| |  | | |
| DISCUSSION |  | | |
| \| Interpretation/ scientific implications \| 18 \| a. The present study has demonstrated that oral administration of adenine at 50 mg/kg for 28 days in mice and at 600 mg/kg for 10 days in rats induces severe anemia, which is accompanied by renal tissue injury and dysfunction. Therefore, we have established a novel approach to develop renal anemic models in rodents. These animal models would be important and useful tools for translational research on renal anemia in humans. \| \| --- \| --- \| --- \| |  | | |
| \| Generalisability/ translation \| 19 \| As adenine specifically induces renal injury, in other animals by oral dosing adjusting with body weight, it would be possible to generate severe anemia. \| \| --- \| --- \| --- \| |  | | |
| \| Funding \| 20 \| This work was supported by the Japan Society for the Promotion of Science (JSPS) Grants-in-Aid for Scientific Research (KAKENHI) (26460343 to Akira Nishiyama) and the Hoansha Foundation (to Akira Nishiyama). \| \| --- \| --- \| --- \| | |  |  |


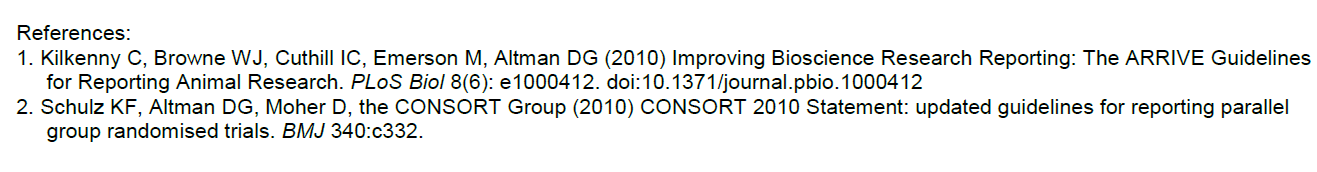

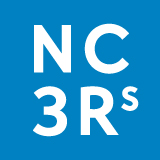

Supplement: S1 File — (DOCX) [file pone.0192531.s003.docx]
